# Functional, radiological and biological markers of alveolitis and infections of the lower respiratory tract in patients with systemic sclerosis

**DOI:** 10.1186/1465-9921-6-96

**Published:** 2005-08-17

**Authors:** Maria De Santis, Silvia Bosello, Giuseppe La Torre, Anna Capuano, Barbara Tolusso, Gabriella Pagliari, Riccardo Pistelli, Francesco Maria Danza, Angelo Zoli, Gianfranco Ferraccioli

**Affiliations:** 1 Department of Rheumatology, Institute of Internal Medicine and Geriatrics, Catholic University of the Sacred Heart, 00168 Rome, Italy; 2 Unit of Epidemiology and Biostatistics, Institute of Hygiene, Catholic University of the Sacred Heart, 00168 Rome, Italy; 3 Department of Pulmonary Medicine, Institute of Internal Medicine and Geriatrics, Catholic University of the Sacred Heart, 00168 Rome, Italy; 4 Institute of Radiology, Catholic University of the Sacred Heart, 00168 Rome, Italy

## Abstract

**Background:**

A progressive lung disease and a worse survival have been observed in patients with systemic sclerosis and alveolitis. The objective of this study was to define the functional, radiological and biological markers of alveolitis in SSc patients.

**Methods:**

100 SSc patients (76 with limited and 24 with diffuse disease) underwent a multistep assessment of cardiopulmonary system: pulmonary function tests (PFTs) every 6–12 months, echocardiography, high resolution computed tomography (HRCT) and bronchoalveolar lavage (BAL), if clinically advisable. Alveolar and interstitial scores on HRCT and IL-6 plasma levels were also assessed as lung disease activity indices.

**Results:**

90 SSc patients with abnormal PFTs and 3 with signs and/or symptoms of lung involvement and normal PFTs underwent HRCT and echocardiography. HRCT revealed evidence of fibrosis in 87 (93.5%) patients, with 55 (59.1%) showing both ground glass attenuation and fibrosis. In 42 patients who had exhibited ground glass on HRCT and consented to undergo BAL, 16 (38.1%) revealed alveolitis. 12 (75%) of these patients had restrictive lung disease (p < 0.0001) and presented diffuse skin involvement (p = 0.0009). IL-6 plasma levels were higher in patients with alveolitis than in patients without (p = 0.041). On logistic regression model the best independent predictors of alveolitis were diffuse skin involvement (OR(95%CIs):12.80(2.54–64.37)) and skin score > 14 (OR(95%CIs):7.03(1.40–34.33)). The alveolar score showed a significant correlation with IL-6 plasma levels (r = 0.36, p = 0.001) and with the skin score (r = 0.33, p = 0.001). Cultures of BAL fluid resulted positive in 10 (23.8%) of the 42 patients that underwent BAL and after one year a deterioration in PFTs occurred in 8 (80%) of these patients (p = 0.01). Pulmonary artery systolic pressure ≥ 40 mmHg was found in 6 (37.5%) patients with alveolitis.

**Conclusion:**

We found alveolitis only in 38.1% of the patients who had exhibited ground glass on HRCT and then underwent BAL, probably because the concomitant fibrosis influenced results. A diffuse skin involvement and a restrictive pattern on PFTs together with ground glass on HRCT were judged possible markers of alveolitis, a BAL examination being indicated as the next step. Nevertheless BAL would be necessary to detect any infections of the lower respiratory tract that may cause further deterioration in lung function.

## Background

There are two types of lung disease in systemic sclerosis (SSc): pulmonary interstitial fibrosis and pulmonary arterial hypertension (PAH) [1]. Additionally there are two subgroups of SSc patients with interstitial lung disease: patients whose lung function deterioration is either stable or shows slow progress and patients with progressive lung disease, frequent secondary vascular involvement and worse survival. Bouros et al reported in a large cohort of SSc patients that the most common histopathologic patterns of SSc lung, non specific interstitial pneumonia (NSIP) and usual interstitial pneumonia (UIP), showed few differences in five-year survival and concluded that the outcome in SSc patients was linked more closely to disease severity at presentation than to histopathologic findings [2]. Morgan et al reported the prognostic value of functional lung indices at the onset of disease and pointed out abnormal forced vital capacity (FVC) and diffusion capacity for carbon monoxide (DLCO) in the early stage of SSc as predictors of end-stage lung disease [3]. Bronchoalveolar lavage (BAL) showed a prognostic value in predicting increased mortality in SSc patients and can identify patients with alveolitis before extensive lung disease has developed allowing earlier intervention [4]. In addiction BAL procedure entails fewer risks and lower costs than lung biopsy and it is the recommended method for obtaining specimens from the lower airways [5,6]. It has been reported that BAL quantitative cultures can discriminate between subjects with and without lung infection with a power comparable or superior to all of the commonly accepted diagnostic tests [6].

We applied a multistep approach to define cardiopulmonary involvement in a cohort of 100 SSc patients: pulmonary function tests (PFTs) every 6–12 months and echocardiography, high resolution computed tomography (HRCT) and bronchoalveolar lavage (BAL) if they are clinically advisable. This study summarises our results in defining a diagnostic approach and analysing the characteristics of patients with alveolitis with the aim of identifying those factors that will potentially act as markers of alveolitis. An alveolar and interstitial score system on HRCT was applied and IL-6 plasma levels were also assessed to better characterize inflammatory and fibrotic lung involvement.

## Methods

### Patients

One hundred (92 females and 8 males) Italian SSc patients attending the outpatient clinic of the Division of Rheumatology of the Catholic University (Rome, Italy) in the last ten years were included in the study. Age (mean ± sd) of the SSc patients was 55.4 ± 11.9 years. The median disease duration was 6 years (range 3–13), duration being calculated as the time from the onset of the first clinical event that was a clear manifestation of SSc (other than Raynaud's phenomenon) to the time of data collection. All patients fulfilled the criteria proposed by the American College of Rheumatology [7], and were grouped according to the classification system proposed by LeRoy et al. [8] in patients with limited or diffuse skin involvement. Modified Rodnan Skin Score was performed for all patients [9]. ANA (antinuclear antibodies) were determined by indirect immunofluorescence using Hep-2 cells as substrates and autoantibodies specificities were further assessed by enzyme-linked immunosorbent assay (ELISA) (Shield, Dundee, UK) [10]. Plasma levels of IL-6 were examined by ELISA method, as described by manufacturer (Biosource, Nivelles Belgium); blood samples were taken from all the patients when HRCT or PFTs were carried out; IL-6 plasma levels in 32 healthy blood donors, matched for age and sex, were 0.31 ± 0.93 pg/ml. Patients were categorized as non-smokers (68%), current smokers (14%) or ex-smokers (18%), ex-smokers being defined as patients who had smoked a minimum of one cigarette a day for a minimum of one year and then stopped at least one year before presentation (Table 1).

**Table 1:** Demographic, clinical and immunological characteristics of 100 SSc patients

	SSc patients
Age (years), mean ± sd	55.4 ± 11.9
	
Disease duration (years), median (I.Q. range)	6 (3–13)
	
Sex n (%)	92 F (92%) 8 M (8%)
Skin involvement:	
dSSc n (%)	24 (24%)
lSSc n (%)	76 (76%)
Autoantibodies pattern:	
ACA n (%)	41 (41%)
AntiScl 70 n (%)	38 (38%)
Antinucleolus n (%)	10 (10%)
AntiRNP n (%)	6 (6%)
ACA/Scl 70/nucleolus/RNP negative n (%)	5 (5%)
	
Smokers n (%)	14 (14%)
Ex-smokers n (%)	18 (18%)

SSc: systemic sclerosis; dSSc: diffuse skin involvement; lSSc: limited skin involvement; ACA: anticentromere antibodies; antiScl 70: antitopoisomerasi I antibodies; antiRNP: antiribonucleoproteins antibodies.

### Therapy

All patients received Iloprost (by an infusion of 0.5–2 ng/kg/minute, lasting 6 hours, for 5 days every six months), Ca-channel blockers (nifedipine 20–40 mg/day) and D-Penicillamine (150 mg/day) from the moment of the medical diagnosis. A wash out period of three months elapsed between an Iloprost course and either PFTs or echocardiography or BAL.

### Pulmonary function tests

All SSc patients underwent PFTs every 6–12 months. PFTs were performed to define FVC and DLCO. FVC was measured using a light bell spirometer in sitting patients wearing nose clip. DLCO was measured using the single breath technique, with 10 seconds breath holding time. All measurements were performed according to the American Thoracic Society recommendations [11] and expressed as percent of predicted values based on age, sex and height [12-14]. Lung involvement evaluated with PFTs was defined as "normal" when FVC and DLCO were ≥ 80%, "mild" when FVC was 70–79% / DLCO was 70–79%, "moderate" when FVC was 50–69% / DLCO was 50–69% and "severe" when FVC was < 50% / DLCO was < 50%, using the assessment of disease severity and prognosis of SSc patients proposed by Medsger et al [15]. Clinically significant restrictive lung disease was defined when an abnormal FVC with normal FEV1/FVC was observed. At one year follow-up we considered a reduction in FVC and/or DLCO >10% as a deterioration in PFTs.

### High resolution computed tomography score system

Patients with abnormal PFTs and/or signs or symptoms of lung involvement (persistent cough, dyspnea on exertion, low degree fever or bilateral crackles) underwent HRCT and echocardiography. PFTs, echocardiography and HRCT examinations were performed in a time frame of three months each from the others. HRCT was performed with 1.0 mm thick sections taken at 10 mm intervals throughout the entire thorax and reconstructed using a spatial frequency algorithm. All images were obtained at the suspended end-inspiratory volume in the supine position. In a limited number of cases showing opacity in the postero-basal segments, sections were acquired also with the patient prone, to ensure that results were not affected by gravity. Three independent readers scored ground glass opacity (alveolar score) and honeycombing (interstitial score) as reported by Kazerooni et al [16] on a scale of 0–5 in the three lobes of both lungs as follows: 0- no alveolar disease, 1- ground glass involving < 5% of the lobe, 2- ground glass involving up to 25% of the lobe, 3- ground glass involving 25–49% of the lobe, 4- ground glass involving 50–75% of the lobe, 5- ground glass involving > 75% of the lobe for alveolar score; 0- no interstitial disease, 1- septal thickening without honeycombing, 2- honeycombing involving up to 25% of the lobe, 3- honeycombing involving 25–49% of the lobe, 4- honeycombing involving 50–75% of the lobe, 5- honeycombing involving > 75% of the lobe for interstitial score. Each observer assessed the extent of involvement in each of 3 defined regions: above aortic arch, between arch and inferior pulmonary veins and between inferior pulmonary veins and lung base. The mean estimate of the three readers was used to define the interstitial and alveolar score for each lobe. The scores were also summed into an overall interstitial and alveolar score. Alveolar or interstitial score ≥ 2 was used to define lung involvement.

### Echocardiography

Pulmonary artery systolic pressure (PASP) was assumed to be equal to the right ventricular systolic pressure (RVSP) when there was no obstruction of the right ventricular outflow. RVSP was calculated with the simplified Bernoulli equation using the maximum peak of tricuspid valve regurgitation velocity (V) and right atrial pressure (RAP) assumed to be 10 mmHg (RVSP = 4V^2^+RAP). To account for the expected increase in PASP with aging, PAH was considered present if PASP exceeded 40 mmHg [17]. PAH was considered secondary to interstitial lung disease in patients with restrictive pattern on PFTs and/or interstitial score ≥ 2 in at least one lobe on lung HRCT.

### Bronchoalveolar lavage analysis

If patients had FVC and/or DLCO ≤ 79% and an alveolar score ≥ 2, they were asked to consent to a BAL examination. The site chosen was generally the one that appeared the most affected on the HRCT analysis. BAL was performed under topical anesthesia (lidocaine 2%, 5–10 ml) without premedication. Four 60 ml aliquots of saline fluid (37°C) were sequentially instilled. Total cell count was performed in a Burker chamber using an uncentrifuged specimen and the result expressed as cells/ml of recovered fluid. BAL fluid was cytocentrifuged for 5 minutes at 500 rpm and differential cell count was performed by light microscope examination of 500 nonephitelial cells after staining with May-Grunwald-Giemsa. The proportions of alveolar macrophages, lymphocytes, neutrophils and eosinophils were recorded. Alveolitis was diagnosed when the percentage of lymphocytes was ≥ 15% and/or neutrophils ≥ 5% and/or eosinophils ≥ 5% [18]. The first aliquot of BAL fluid was used for microbiological studies (bacteria, mycobacteria, fungi, parasites) [5]. 10^4 ^colony forming units/ml of BAL were considered significant amounts of bacterial growth [6].

### Statistical analysis

Data were analyzed using SPSS 11.0 (SPSS. Chicago. IL-USA) and Prism software (Graph-Pad, S. Diego, CA 92121-USA). Categorical and quantitative variables were respectively described as numbers, percentage (%) and mean ± standard deviation (sd) or median and I.Q. range, according to data distribution. Mann-Whitney's test was used to compare continuous variable. Categorical variables were analysed using χ^2 ^test or Fisher's test, depending on sample size restrictions and the Odd ratios (OR) with 95% confidence interval (95% CIs) were calculated. Spearman's rank correlation was used to evaluate the relationship between different disease parameters. A logistic regression model was used in order to determine the influence on the dependent variable "having alveolitis" by the independent variables that reached the value of p <0.25 at the univariate analysis. The values are expressed as OR (95% CI). The diagnostic values of the clinical variables were assessed by calculating the areas under the receiver operating characteristics (ROC) curves, which were used to assess the best cut-off points to identify the presence of alveolitis. The diagnostic accuracy was calculated by sensitivity and specificity. We used a stepwise procedure (backward elimination), following the method suggested by Hosmer and Lemeshow. The chi-square test and the Hosmer-Lemeshow test were used in order to assess the fitting of the model. A value of p < 0.05 was considered statistically significant.

## Results

### Clinical and immunological data

76 (76.0%) patients had limited (lSSc) and 24 (24.0%) diffuse skin involvement (dSSc). Anticentromere (ACA) was present in 41 patients (41.0%), antitopoisomerasi I (antiScl 70) in 38 (38.0%). 10 (10.0%) patients were antinucleolus positive, 6 (6.0%) were antiribonucleoproteins (antiRNP) positive and 5 (5.0%) were ACA/antiScl 70/antinucleolus/antiRNP negative (Table 1).

### PFTs results

PFTs results showed that patients could be divided into 3 groups: patients with restrictive pattern, patients with isolated reduction in DLCO and patients with normal PFTs. Characteristics of the 3 groups are detailed in Table 2. 16 (16.0%) patients had FVC and DLCO ≤ 79%: 12 of these patients had FVC and DLCO ≤ 69% and 1 had FVC and DLCO ≤ 49%; 74 (74.0%) patients had FVC > 80% and DLCO ≤ 79%: 67 of these patients had DLCO ≤ 69% and 16 had DLCO ≤ 49%; 10 (10.0%) patients had normal PFTs. FVC and DLCO ≤ 79% was observed in 12 (50%) of the 24 dSSc patients and in 10 (41.7%) of these patients FVC and DLCO ≤ 69% occurred. Of the 76 lSSc patients, FVC and DLCO ≤ 79% was observed in 4 (5.3%) and FVC and DLCO ≤ 69% occurred in 2 (2.6%) (data not reported in table).

**Table 2:** PFTs results of 100 SSc patients

	FVC ≤ 79% DLCO ≤ 79% 16 patients	FVC ≥ 80% DLCO ≤ 79% 74 patients	FVC ≥ 80% DLCO ≥ 80% 10 patients
Age (years), mean ± sd	55.1 ± 12.4	56.1 ± 11.9	50.7 ± 11.4
			
Disease duration (years), median (I.Q. range)	7 (4 – 18)	6 (3 – 13)	4.5 (1 – 14.5)
			
Sex n (%)	14 F (87.5%) 2 M (12.5%)	68 F (91.9%) 6 M (8.1%)	10 F (100.0%) 0 M (0.0%)
Skin involvement:			
dSSc n (%)	12 (75.0%)	12 (16.2%)	0 (0.0%)
lSSc n (%)	4 (25.0%)	62 (83.8%)	10 (100.0%)
Skin score mean ± sd	14.8 ± 10.7	9.9 ± 7.4	6.7 ± 4.6
Autoantibodies pattern:			
ACA n (%)	2 (12.5%)	32 (43.2%)	7 (70.0%)
AntiScl 70 n (%)	11 (68.7%)	25 (33.8%)	2 (20.0%)
Antinucleolus n (%)	0 (0.0%)	9 (12.2%)	1 (10.0%)
AntiRNP n (%)	1 (6.3%)	5 (6.8%)	0 (0.0%)
ACA/Scl 70/nucleolus/RNP negative n (%)	2 (12.5%)	3 (4.0%)	0 (0.0%)
FVC (%) mean ± sd	65.0 ± 8.8	104.8 ± 16.3	122.0 ± 13.3
≤ 69% n (%)	12 (75.0%)	0 (0.0%)	0 (0.0%)
≤ 49% n (%)	1 (6.3%)	0 (0.0%)	0 (0.0%)
DLCO (%) mean ± sd	39.8 ± 11.8	59.0 ± 10.4	94.2 ± 12.6
≤ 69% n (%)	16 (100%)	67 (90.5%)	0 (0.0%)
≤ 49% n (%)	13 (81.3%)	16 (21.6%)	0 (0.0%)
FVC and DLCO ≤ 69%	12 (75.0%)	0 (0.0%)	0 (0.0%)
FVC and DLCO ≤ 49%	1 (6.3%)	0 (0.0%)	0 (0.0%)
PASP ≥ 40 mmHg n (%)	6 (37.5%)	5 (6.7%)	0 (0.0%)
≥ 60 mmHg n (%)	1 (6.3%)	1 (1.3%)	0 (0.0%)

PFTs: pulmonary function tests; FVC: forced vital capacity; DLCO: diffusing capacity for carbon monoxide; dSSc: diffuse skin involvement; lSSc: limited skin involvement; ACA: anticentromere antibodies; antiScl 70: antitopoisomerasi I antibodies; antiRNP: antiribonucleoproteins antibodies; PASP: pulmonary artery systolic pressure.

### Functional, radiological and BAL analysis

90 patients with abnormal PFTs and 3 patients with clinical signs and/or symptoms of lung involvement and normal PFTs underwent echocardiography and HRCT (Table 3). PASP ≥ 40 mmHg was found in 11 (11.8%) patients, 2 patients had PASP ≥ 60 mmHg. In 7 (7.5%) patients elevated PASP was considered secondary to interstitial lung disease (see also Additional file 1). HRCT revealed evidence of fibrosis in 87 (93.5%) patients, while 55 (59.1%) patients had both ground glass attenuation and fibrosis. 42 patients, whose HRCT had exhibited ground glass attenuation, gave their informed consent to a BAL examination. Alveolitis, detected by the BAL cell criteria as reported above, was found in 16 (38.1%) of these patients. Percentage of neutrophils was ≥ 5% in all 16 patients with alveolitis. In 2 cases there was also a percentage of lymphocytes ≥ 15%, in 2 more cases percentage of eosinophils ≥ 5% was also present and in 1 case the percentage of the three cell lines was increased. Cultures of BAL fluid resulted positive in 10 (23.8%) patients. Microbiological analysis revealed: Candida in 3 cases, Candida, Aspergillus and Stenotrophomonas in 1 case, Candida and Haemophilus in 1 case, Haemophilus in 1 case, Fusarium oxysporium in 1 case, Neisseria in 1 case, Enterococcus faecalis in 1 case, Streptococcus pneumoniae in 1 case.

**Table 3:** Functional, radiological and BAL analysis.

	100 SSc patients
FVC % mean ± sd	100.1 ± 22.1
≤ 79% n (%)	16 (16.0%)
≤ 69% n (%)	12 (12.0%)
≤ 49% n (%)	1 (1.0%)
DLCO % mean ± sd	59.4 ± 17.3
≤ 79% n (%)	90 (90.0%)
≤ 69% n (%)	83 (83.0%)
≤ 49% n (%)	29 (29.0%)
PASP ≥ 40 mmHg n (%)*	11 (11.8%)*
≥ 60 mmHg n (%)*	2 (2.2%)*
HRCT: interstitial score ≥ 2, n (%)*	87 (93.5%)*
mean total score ± sd	3.1 ± 4.2
HRCT: alveolar score ≥ 2, n (%)*	55 (59.1%)*
mean total score ± sd	5.7 ± 2.6
BAL: alveolitis n (%)**	16 (38.1%)**
N ≥ 5 % n (%)**	11 (68.8%)**
N ≥ 5 % and L ≥ 15 % n (%)**	2 (12.5%)**
N ≥ 5 % and E ≥ 5 % n (%)**	2 (12.5%)**
N ≥ 5 %, L ≥ 15 % and E ≥ 5 % n (%)**	1 (6.2%)**
N < 5 % and L ≥ 15 % and/or E ≥ 5 % n (%)**	0 (0.0%)**
BAL with positive cultures n (%)**	10 (23.8%)**

BAL: bronchoalveolar lavage; SSC: systemic sclerosis; FVC: forced vital capacity; DLCO: diffusing capacity for carbon monoxide; PASP: pulmonary artery systolic pressure; HRCT: high resolution computed tomography; N: neutrophils; L: lymphocytes; E: eosinophils.

*Echocardiography and HRCT performed in 93 SSc patients

** BAL performed in 42 SSc patients giving their informed consent

### Characteristics of patients with alveolitis

Patients with alveolitis did not show significant differences in demographic characteristics and autoantibodies when compared to patients without alveolitis (Table 4). Diffuse skin involvement was present in 12 (75.0%) patients with alveolitis: patients with diffuse skin involvement and ground glass attenuation on HRCT were at higher risk of having alveolitis than patients with limited skin disease and ground glass with an odd ratio of 12.60 (95% CIs = 2.83–56.15, p = 0.0009 Fisher's test). Moreover the mean modified Rodnan skin score was higher in patients with alveolitis than in patients without (p = 0.038). Modified Rodnan skin score > 14 [9] was observed in 8 (50.0%) patients with alveolitis and in 5 (19.2%) without alveolitis (p = 0.036).

**Table 4:** Characteristics of patients with alveolitis

	BAL: alveolitis 16 patients	BAL: inactive 26 patients	P
Age (years) mean ± sd	57.0 ± 9.8	57.1 ± 11.8	ns
Disease duration (years) median (I.Q. range)	7.5 (3–16)	5 (1.5–12)	ns
Sex n (%)	1 (6.25%)	2 (7.7%)	ns
Autoantibodies pattern:			
ACA n (%)	2 (12.5%)	8 (30.8%)	ns
AntiScl 70 n (%)	12 (75.0%)	11 (42.3%)	ns
Antinucleolus n (%)	0 (0.0%)	4 (15.4%)	ns
AntiRNP n (%)	1 (6.25%)	1 (3.8%)	ns
ACA/Scl 70/nucleolus/RNP negative n (%)	1 (6.25%)	2 (7.7%)	ns
Skin involvement:			
dSSc n (%)	12 (75.0%)	5 (19.2%)	0.0009
lSSc n (%)	4 (25.0%)	21 (80.8%)	0.0009
Modified Rodnan Skin Score mean ± sd	16.3 ± 9.4	10.1 ± 9.1	0.038
Modified Rodnan Skin score >14 n (%)	8 (50.0%)	5 (19.2%)	0.036
PFTs: FVC mean (%) ± sd	76.1 ± 27.6	101.9 ± 17.0	0.0005
DLCO mean (%) ± sd	43.9 ± 16.6	54.8 ± 11.0	0.0086
FVC and DLCO ≤ 79% n (%)	12 (75.0%)	2 (7.7%)	< 0.0001
FVC and DLCO ≤ 49% n (%)	10 (62.5%)	2 (7.7%)	< 0.0001
FVC ≥ 80% and DLCO ≤ 79% n (%)	3 (18.8%)	24 (92.3%)	< 0.0001
FVC ≥ 80% and DLCO ≤ 49% n (%)	10 (62.5%)	9 (34.6%)	ns
FVC and DLCO ≥ 80% n (%)	1 (6.3%)	0 (0.0%)	-
PASP ≥ 40 mmHg n (%)	6 (37.5%)	1 (3.8%)	0.008
≥ 60 mm Hg n (%)	1 (6.3%)	0 (0.0%)	-
HRCT: interstitial score mean ± sd	6.9 ± 3.5	7.3 ± 1.9	ns
alveolar score mean ± sd	9.1 ± 5.3	5.0 ± 3.1	0.0095
C reactive protein mean (mg/l) ± sd	8.6 ± 10.8	6.6 ± 6.7	ns
IL-6 plasma levels (pg/ml) mean ± sd	6.0 ± 10.8	2.4 ± 4.1	0.041
BAL with positive microbiological culture n (%)	5 (31.3%)	5 (19.2%)	ns
Smokers n (%)	1 (6.3%)	4 (15.4%)	ns
Ex-smokers n (%)	3 (18.8%)	5 (19.2%)	ns

BAL: bronchoalveolar lavage; ACA: anticentromere antibodies; antiScl 70: antitopoisomerasi I antibodies; antiRNP: antiribonucleoproteins antibodies; dSSc: diffuse skin involvement; lSSc: limited skin involvement; FVC: forced vital capacity; DLCO: diffusing capacity for carbon monoxide; PASP: pulmonary artery systolic pressure; HRCT: high resolution computed tomography.

The mean FVC (%) and the mean DLCO (%) were significantly lower in patients with alveolitis (p = 0.0005 for FVC, p = 0.0086 for DLCO, respectively *vs *patients without alveolitis), moreover alveolitis was found in 12 (75.0%) patients with restrictive pattern on PFTs (p < 0.0001 *vs *patients with restrictive pattern but without alveolitis) and in 3 (18.8%) patients with an isolated reduction in DLCO that underwent BAL. One patient with normal PFTs underwent HRCT because of a persistent cough: BAL, assessed because of the presence of ground glass on HRCT, revealed alveolitis. Data on PFTs one year before BAL examination were available only for 10 patients with subsequent diagnosis of alveolitis and for 15 patients without alveolitis. However there were no differences in the percentage of patients with a clinically significant reduction in FVC or DLCO.

The mean alveolar score was significantly higher (9.1 ± 5.3) in patients with alveolitis *vs *patients without (5.0 ± 3.1; p = 0.0095), while no differences were seen in the mean interstitial score (p = ns). PASP ≥ 40 mmHg was found in 6 (37.5%) patients with alveolitis and in 1 (3.8 %) patients without (p = 0.008); in 1 (6.3%) patient PASP was ≥ 60 mmHg.

C reactive protein (CRP) values tended to be higher in patients with alveolitis (8.6 ± 10.8 mg/l) than in patients without alveolitis (6.6 ± 6.7 mg/l), but the difference between the two groups was not statistically significant. IL-6 plasma levels were significantly higher in patients with alveolitis (6.0 ± 10.8 pg/ml) than in patients without (2.4 ± 4.1 pg/ml; p = 0.041).

Skin score (>14), skin involvement extent, autoantibodies pattern, FVC (≤ 79%), DLCO (≤ 49%), alveolar score on HRCT (> 6), IL-6 plasma levels (> 0.75 pg/ml) and CRP (> 5 mg/l) were the components of the logistic regression model for alveolitis. The cut-off values of the dependent variables considered in the analysis were based on ROC curves and are reported in table 5. The best independent predictors of alveolitis were the skin score > 14 (OR (95%CIs): 7.03 (1.4–34.33)) and diffuse skin involvement (OR(95%CIs): 12.80 (2.54–64.37)). If the skin involvement was excluded from the model IL-6 was seen to be the best independent marker of alveolitis (OR (95%CIs): 6.22 (1.37–38.37)).

**Table 5:** Diagnostic accuracy of the predictors of alveolitis

	AUC (95% CI)	p	Cut-off value	Se(%)	Sp (%)
FVC	0.145 (-0.003 – 0.292)	0.000	80.5	77.8	100.0
DLCO	0.199 (0.058 – 0.340)	0.001	50.0	72.2	70.8
IL-6	0.694 (0.524 – 0.864)	0.045	0.75	80.0	60.9
Skin score	0.650 (0.473 – 0.826)	0.109	14.5	58.8	78.3
Alveolar score on HRCT	0.742 (0.591 – 0.893)	0.008	6.5	66.7	75.0

AUC: area under curve ROC; FVC: forced vital capacity; DLCO: diffusing capacity for carbon monoxide; HRCT: high resolution computed tomography.

The microbiological analysis of BAL fluid was positive in 5 (31.3%) of the patients with alveolitis and in 5 (19.2%) of the patients without (p = ns). IL-6 plasma levels were higher in patients with positive BAL fluid cultures (5.2 ± 4.8 pg/ml) than in patients without infections (3.3 ± 7.8 pg/ml; p = 0.003), and a similar trend was found in CRP serum levels (12.6 ± 13.0 mg/l *vs *5.7 ± 5.7 mg/l; p = ns). Patients with positive BAL fluid cultures showed a deterioration in PFTs after one year in 8 (80.0%) cases (4 with alveolitis and 4 without, p = ns) *vs *10 (31.3%) patients without infection (3 with alveolitis and 7 without, p = ns) despite antimicrobial treatment, thus suggesting that infection is a poor prognostic factor (OR(95% CIs):8.8 (1.60–49.02), p = 0.01). Instead deterioration in PFTs after one year was observed in 7 (43.8%) patients with alveolitis *vs *11 (42.3%) patients without (p = ns).

Patients with ground glass on HRCT that refused the BAL examination (13 patients) did not show significant differences in demographic, clinical and functional features when compared to patients who underwent BAL; alveolar and interstitial scores, IL-6 plasma levels and CRP were lower in patients that did not perform BAL (see Additional file 2).

### HRCT scores correlations

**Figure 1:**
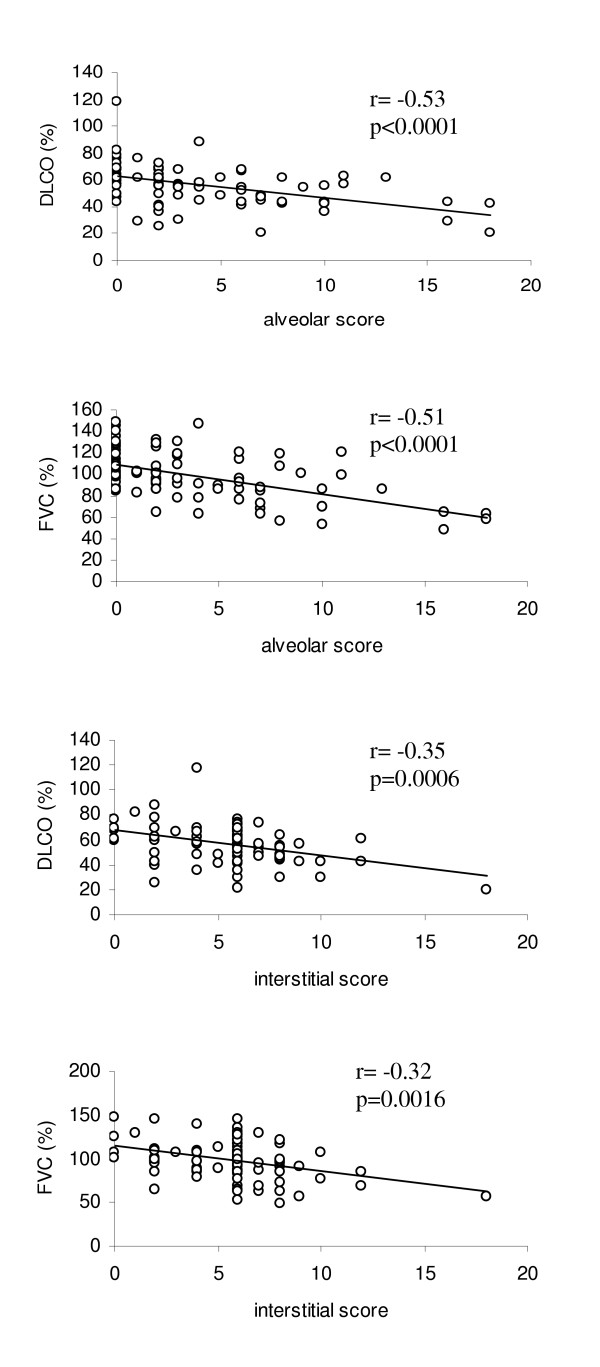
**Correlations between HRCT scores and PFTs in SSc patients**. FVC showed a significant correlation with both the alveolar score (r = -0.51, p < 0.0001) and the interstitial score (r = - 0.32, p = 0.0016). Similarly DLCO showed a significant correlation with the alveolar score (r = -0.53, p < 0.0001) and the interstitial score (r = -0.35, p = 0.0006).

**Figure 2:**
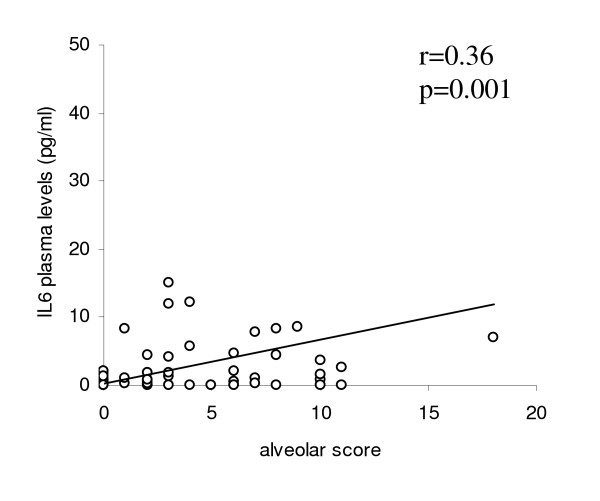
**Correlation between alveolar score and IL-6**. The alveolar score showed a statistically significant correlation with IL-6 plasma levels (r = 0.36, p = 0.0012).

**Figure 3:**
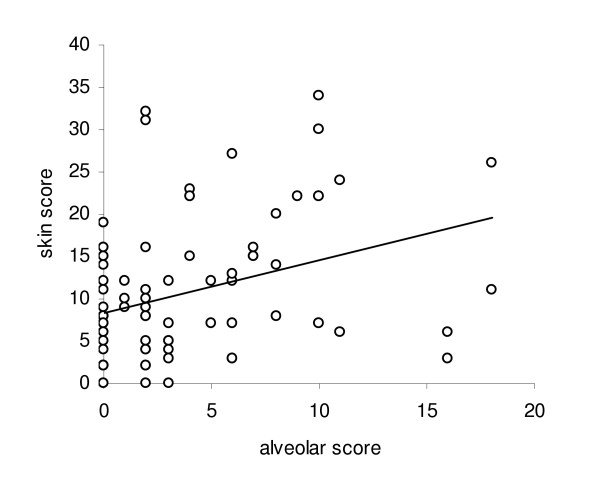
**Correlation between alveolar score and skin score**. The alveolar score showed a statistically significant correlation with the skin score (r = 0.33, p = 0.0021).

We found a correlation between alveolar and interstitial scores, assessed by the HRCT score system proposed by Kazerooni et al, and FVC (r = -0.51, p < 0.0001 for alveolar score; r = -0.32, p = 0.0016 for interstitial score, respectively) and DLCO (r = -0.53, p < 0.0001 for alveolar score; r = -0.35, p = 0.0006 for interstitial score, respectively) (Figure 1). In addiction, the alveolar score showed a statistically significant correlation with IL-6 plasma levels (r = 0.36, p = 0.0012) and skin score (r = 0.33, p = 0.0021) (Figure 2 and 3 respectively).

## Discussion

Detecting alveolitis is an important diagnostic clue in assessing disease severity in SSc patients. A greater deterioration in pulmonary function, a larger extent of lung fibrosis on HRCT over time and an increased mortality have been reported in patients with untreated alveolitis [4,19]. Great differences in the prevalence of alveolitis have been noted in past studies (from 48% to 72%), even considering those with a high number of patients [4,19-21] probably because of the different ratios of patients with diffuse and limited disease. Moreover no clear correlation has been reported between lung function indices, ground glass on HRCT and the presence of alveolitis, even though patients with alveolitis seemed to have worse FVC and DLCO than patients without [20-22] and it has long been demonstrated that ground glass attenuation on HRCT is the probable result of an inflammatory process [23-25].

Abnormal PFTs, especially a decreased DLCO, are a common finding in SSc patients: 90% of our cohort had abnormal DLCO with or without a decrease in FVC. Clinically significant restrictive lung disease was seen only in 16% of our cohort but in 50% of patients with diffuse skin involvement, while moderate-severe restrictive lung disease occurred in 12% of the whole cohort and in 41.7% of the patients with diffuse skin involvement. Despite differences between cohorts, this is confirmed by previous studies: the prevalence of restrictive lung disease among SSc patients varies between 25% and 35%, and between 30% and 70% in patients with diffuse disease [20,26-29]. In our study PASP ≥ 40 mmHg was found in 11.8% of the patients with abnormal PFTs and/or signs or symptoms of lung involvement: in 7.5% of cases elevated PASP could be secondary to interstitial lung disease, less than reported in a large cohort of scleroderma patients in which a restrictive ventilatory defect was observed in 22% of the patients and secondary PAH in 18% of the patients [30]. In our cohort 93.5% of patients with abnormal PFTs had an interstitial score ≥ 2 on HRCT and 59.1% showed an alveolar score ≥ 2, but only 38.1% of the patients that underwent BAL because of ground glass attenuation on HRCT had alveolitis defined by the BAL cell criteria detailed above. In our study all patients with ground glass attenuation showed concomitant signs of fibrosis on HRCT, as previously reported in a significant percentage of SSc patients [31]. Ground glass attenuation with concomitant signs of fibrosis, such as traction bronchiectasis or a reticular pattern, does not always lead to the identification of an inflammatory process and this could explain our data. It has been reported that fine intralobular fibrosis increases lung density on HRCT resulting in ground glass attenuation that is indistinguishable from the HRCT appearance found in alveolitis or in any other inflammatory process which results in accumulation of inflammatory cells or oedema in the alveolar septa and air spaces, as occurs in infections [32]. In addiction in SSc lung, especially in the early stages of the disease, histopathologic studies have shown hypercellularity of the alveolar wall [33], oedema [34] and over-development of microvessels that are abnormal in both shape (multi-bubbles and intervascular fusion) and size in the alveolar septa and interstitium [35] resulting in increased capillary blood volume which could also cause ground glass attenuation [32].

The functional significance of the two major radiographic patterns of interstitial lung involvement, ground glass and fibrosis, has not been clarified in previous studies. No relation has been found between HRCT findings and parameters of disease severity, such as a decrease in DLCO and FVC [36] or biological markers. Moreover, when the extent of lung involvement was assessed without distinguishing ground glass and reticular pattern on HRCT, no relationship with parameters of lung function was found, except with DLCO [37,38]. This suggests that DLCO fails to discriminate between inflammatory and fibrotic lung involvement. Instead, when the extent of ground glass and fibrotic patterns were assessed separately, an inverse correlation with FVC and DLCO was found, as reported by Ooi et al [29]. Similar results were found in our study where the interstitial score and the alveolar score were assessed as described by Kazerooni et al [16]. The alveolar score also showed a significant correlation with the modified Rodnan skin score and IL-6 plasma levels, thus suggesting that patients with a greater extent of ground glass attenuation on HRCT had a more aggressive disease. In addition, higher IL-6 plasma levels suggest that inflammation could explain the more aggressive pulmonary disease in patients with alveolitis. Previous studies have identified IL-6 serum levels as a useful index of disease activity in SSc patients because of the correlation with the skin score and suggested a pathogenic role of IL-6 in skin fibrosis [39,40]. In our study the significant correlation between IL-6 plasma levels and alveolar score and the higher values of IL-6 found in patients with alveolitis seem to confirm the helpful role of IL-6 as a disease activity index. Further studies could clarify the IL-6 role in inflammatory pulmonary involvement in SSc patients.

When PFTs are abnormal, HRCT is therefore an essential second step to assess the extent of interstitial disease and to detect the presence of inflammation in SSc lung involvement but the presence of ground glass alone can identify alveolitis in less than 40% of cases. In our study patients with diffuse skin involvement and ground glass attenuation on HRCT were twelve times more likely to have alveolitis compared to patients with limited skin disease. Moreover 75% patients with alveolitis presented restrictive lung disease. In the logistic regression model the extent of skin involvement appeared as the best predictor of alveolitis. In fact when restrictive pattern on PFTs was considered together with severe reduction in DLCO (≤ 49%), the association with alveolitis disappeared.

Patients with alveolitis showed a more aggressive lung disease as indicated by a worse lung function, a greater extent of pulmonary involvement on HRCT and a higher frequency of PAH. Nevertheless a greater deterioration in pulmonary function at the one year follow-up was observed in patients with positive BAL fluid cultures. In a normal host recovering an infectious agent from the lower respiratory tract does not necessarily mean infection although the recovery of certain organisms is believed to be almost always pathologic and quantitative criteria for bacterial BAL fluid cultures interpretation are standardized [41]. Nevertheless infections of the lower respiratory tract constitute a risk factor for deterioration of pulmonary function especially in patients with interstitial lung disease and may be under-diagnosed in SSc patients with lung involvement. In a few studies BAL fluid cultures were performed and specific infectious agents have been reported [21]. In our cohort 23.8% of the patients that underwent BAL had positive BAL fluid cultures and in 50% of cases fungi or polymicrobial colonization were found. Since BAL has the highest sensitivity for detecting deep-seated fungal infections, quantitative culture techniques have not been investigated and it has not always been possible to distinguish colonization from infection when clinical signs and symptoms are not specific [42]. Antigen tests and PCR test on BAL samples will aid the diagnosis of infections of the lower respiratory tract [42]. Nevertheless, patients with positive BAL fluid cultures seems to be at high risk of faster lung function deterioration, as observed at the one year follow-up in our study. These data probably indicate that even colonization by infectious agents may be a risk factor in worsening of patients with interstitial lung disease or that colonization/infection is present in those patients who are more likely to get worse. Higher IL-6 plasma levels in patients with positive cultures suggest that inflammation could be the worsening factor.

## Conclusion

Considering the limits of functional and radiographic procedures in identifying alveolitis, BAL appears to be an essential tool in characterizing patients at high risk of severe lung disease. In our study not as many patients as expected consented to the BAL examination but despite this, it is one of the largest studies presenting data on PFTs, HRCT score system and BAL examination in systemic sclerosis [2,4,35]. The data obtained lead us to believe that diffuse skin involvement and a restrictive pattern on PFTs together with ground glass on HRCT are possible markers of alveolitis to be followed by a BAL examination. Our data also suggest the importance of detecting infection or colonization of the lower respiratory tract that may lead to an even faster lung function deterioration.

## Competing interests

The author(s) declare that they have no competing interests.

## Authors' contributions

MDS: Conceived the study, participated in the design, performed the study and drafted the manuscript.

SB: Conceived the study, participated in the design, performed the study and helped draft the manuscript.

GLT: Performed statistical analysis.

AC: Participated in the design and performed the study.

BT: Performed laboratory analysis and performed statistical analysis.

GP: Participated in the design and performed the study.

RP: Participated in the design and performed the study.

FMD: Participated in the design and performed the study.

AZ: Participated in the design and helped draft the manuscript.

GF: Conceived the study, participated in the design and co-ordination of the study and helped draft the manuscript.

## Supplementary Material

Additional File 1 **Correlations between PASP and FVC, DLCO and interstitial score. **PASP showed statistically significant correlation with both FVC (r = -0.36, p = 0.006) and DLCO (r = -0.38, p = 0.004) but not with the interstitial score (r = 0.26, p = 0.055). Click here for file

Additional File 2 **Characteristics of patients refusing BAL. **Patients with ground glass on HRCT that refused the BAL examination (13 patients) did not show significant differences in demographic, clinical and functional features when compared to patients who underwent BAL; alveolar and interstitial scores, IL-6 plasma levels and CRP were lower in patients that did not perform BAL. Click here for file
