# Supplementary material for: Functional, radiological and biological markers of alveolitis and infections of the lower respiratory tract in patients with systemic sclerosis
Source: Respir Res. 2005 Aug 17;6(1):96. doi: 10.1186/1465-9921-6-96 (PMC1208954; doi:10.1186/1465-9921-6-96)
Supplement: Additional File 1 — Correlations between PASP and FVC, DLCO and interstitial score. PASP showed statistically significant correlation with both FVC (r = -0.36, p = 0.006) and DLCO (r = -0.38, p = 0.004) but not with the interstitial score (r = 0.26, p = 0.055). [file 1465-9921-6-96-S1.doc]

**Additional file 1: Correlations between PASP and FVC, DLCO and interstitial score**

r=-0.36

p=0.006

r=-0.38

p=0.004

r=0.26

p=0.055

PASP: pulmonary artery systolic pressare; FVC: forced vital capacity; DLCO: diffusing capacity for carbon monoxide.
